# MilkyBase, a database of human milk composition as a function of maternal-, infant- and measurement conditions

**DOI:** 10.1038/s41597-022-01663-1

**Published:** 2022-09-09

**Authors:** Tünde Pacza, Mayara L. Martins, Maha Rockaya, Katalin Müller, Ayan Chatterjee, Albert-László Barabási, József Baranyi

**Affiliations:** 1 Doctoral School of Food and Nutrition Science, Institute of Nutrition, University of Debrecen, Debrecen, Hungary; 2 Heim Pál National Paediatric Institute, Budapest, Hungary; 3 Doctoral School of Clinical Medicine, University of Debrecen, Debrecen, Hungary; 4 Center for Complex Network Research, Northeastern University, Boston, USA; 5 Network Science Institute, Northeastern University, Boston, USA; 6 Department of Medicine, Brigham and Women’s Hospital, Harvard Medical School, Boston, USA; 7 Center for Network Science, Central European University, Budapest, Hungary

**Keywords:** Databases, Data integration

## Abstract

This study describes the development of a database, called MilkyBase, of the biochemical composition of human milk. The data were selected, digitized and curated partly by machine-learning, partly manually from publications. The database can be used to find patterns in the milk composition as a function of maternal-, infant- and measurement conditions and as a platform for users to put their own data in the format shown here. The database is an Excel workbook of linked sheets, making it easy to input data by non-computationally minded nutritionists. The hierarchical organisation of the fields makes sure that statistical inference methods can be programmed to analyse the data. Uncertainty quantification and recording dynamic (time-dependent) compositions offer predictive potentials.

## Abstract (summary)

**Table:** 

Measurement(s)	Concentration of biochemical compounds in human milk or/and derived quantities, like their sums or ratios.
Technology Type(s)	Data mining, by means of Machine Learning and targeted manual literature search within available scientific publications in the internet.
Factor Type(s)	Georgaphical region • Cohort size • Measurement Method • Various characteristics (including history) of mother, child, breast milk and measurement
Sample Characteristic - Organism	Human milk
Sample Characteristic - Environment	Standard birth environment
Sample Characteristic - Location	Various regions of the world

## Background & Summary

The effect of diet on health has primarily been analysed in a descriptive way. Widely acknowledged claims, such as garlic helps preventing cardiovascular diseases, are lacking mechanistic, biochemical explanations^1^. The main sources of such uncertainties are: (i) the complexity caused by thousands of chemical interactions; (ii) the inherent errors in the measurements and observations; (iii) many hitherto unknown other details^1^.

Human milk (HM) is the first nutrition an infant comes across and one of our most complex foods. Ideally, mothers should breastfeed their infants, but we need to acknowledge that in many cases this is not possible, and even when mothers try their best, breastfeeding is challenging and requires a strong supportive environment.

HM has been studied extensively, still its biochemical complexity is insufficiently explored^2,3^. It is the only food that meets all the nutritional requirements of infants and provides optimal adaptation, somatic growth, maturation, and development^4^. Beside the nutrients (carbohydrates, lipids, proteins, vitamins, and minerals), it provides bioactive components (hormones, cytokines, growth factors, antimicrobial substances, cells, etc.), which play important roles in the development of the central nervous system, metabolism, immune system, and microbiome^5–9^. Breastfeeding has been associated with improved health outcomes, including increased intelligence, reduced risks of infections and non-communicable diseases (obesity, atopic diseases, diabetes, inflammatory bowel diseases)^6,7^. This crucial role of HM in early life nutrition gains great clinical^5–7,10^, social and economic interest due to its impact on long-term health^10,11^.

HM is a biological system, where both nutritional and bioactive components are in constant interactions with one another^2^. The exact dynamics depend on characteristics related to the mother, the infant, and various environmental factors (such as the mother’s diet, the gestational age, the geographic location etc.), which are also responsible for the variability of the HM composition^3^. Our current knowledge is largely based on studies evaluating these components, typically analysing their variability and dynamics separately^2,3,7^. Therefore, explaining health outcomes directly by specific components is rarely satisfactory, due to the modifying effects of the interactions between the factors in question^2,3,7^.

As in any complex systems, the dynamics of HM cannot be predicted from the kinetics of its individual components^2,3^. A big-data platform is needed to help. More accurately than ever, an appropriately built database could provide objective, data- and science-based guidance on the diet and lifestyle of lactating women to optimize their children’s health. Besides, the development of HM substitutes could benefit enormously from the collective knowledge the database can store.

In this paper, we demonstrate that an adequately built database, combined with numerical/statistical tools, has huge potentials to unveil food complexity^1,12^ and to benefit from the stored knowledge. A key to this is the basis of our database-building principle: it considers a record as a mapping from various, possibly dynamic explanatory conditions, under which observations have been made, to the composition of HM, a truly dynamic response variable. A vital means to realize this ontology-principle is that the temporal variation of the variables is represented by tables, and pointers to these tables make sure that time-dependence is a natural attribute of the respective fields.

## Methods

Food composition data have already been collected in databases, following various ontology depending on the purpose and the wanted resolution of the database. Our MilkyBase is intended to be used by academia as well as industry and regulation, therefore many compromises had to be made to find a balance between the four-V-principle of Big Data: volume, velocity, veracity, and variety.

### Volume

We have set up a database that hosts published measurements of molecular components of breast milk. With its ca 10,000 datapoints, MilkyBase is far from the volume that is expectable from a Big Data project. However, we hope to initiate an ontology that would be used by researchers as well as clinicians to input their own data, so to create a “periodic table” of other important food-types, as a pool for collective knowledge^13^. Therefore, the template for inputting the data must be user-friendly enough, on commonly used platform, easily handled by the data donors. This is the reason why Microsoft Excel was chosen, as the most ubiquitous package that can link tables and be programmed via the Visual Basic for Applications (VBA) language. The VBA programs will aid both input check and data analysis (such as comparing own and others’ similar observations) and serve as incentives to authors to submit relevant data. This is a kind of wiki-philosophy, which should result in a much bigger data volume than its current size.

### Velocity

With its current size, the navigation and data processing are running at an acceptable speed, but the Excel platform will not be practical as the volume of the data increases, therefore, with time, it will be imported into an SQL server and the Excel sheets will serve a transit area for data donors, for initial curation.

### Variety and veracity

As these are closely related, we discuss them together. Our goal was to digitize published data in a rigorously organized database, ready to be analysed by considering the milk composition as a function of various conditions. Therefore we tried to avoid changing published data, except in trivial cases, such as conversion of units for the sake of uniformity. Many times we found ambiguity or controversy in the terminology used by authors. An example for this is the concentration of a particular fatty acid molecule, which was mostly reported as a proportion relative to the total fatty acid, but sometimes proportion in the total milk mass, and sometimes even just the proportion of the total *measured* fatty acids. In such cases, we used our best knowledge and expert help to make these concepts well-defined and quantified. Such efforts admittedly bear the footprint of the database developer’s judgement.

If there are trivial mistakes in the publication (such as conversion error from one unit to another one) that were easily correctable then we did so; otherwise, either we left the record out, or marked it as “suspicious”. Even so, the resultant database is inevitably imperfect. However, the discrepancies should get detected as the database is being used.

Note that the variety - veracity issue is closely related to the syntax and semantics of the fields of the database. While its syntax can be checked in an automated way, its semantics frequently reveals anomalies, affecting what data can be inputted (variety) and how can those be validated (veracity).

**Fig. 1:**
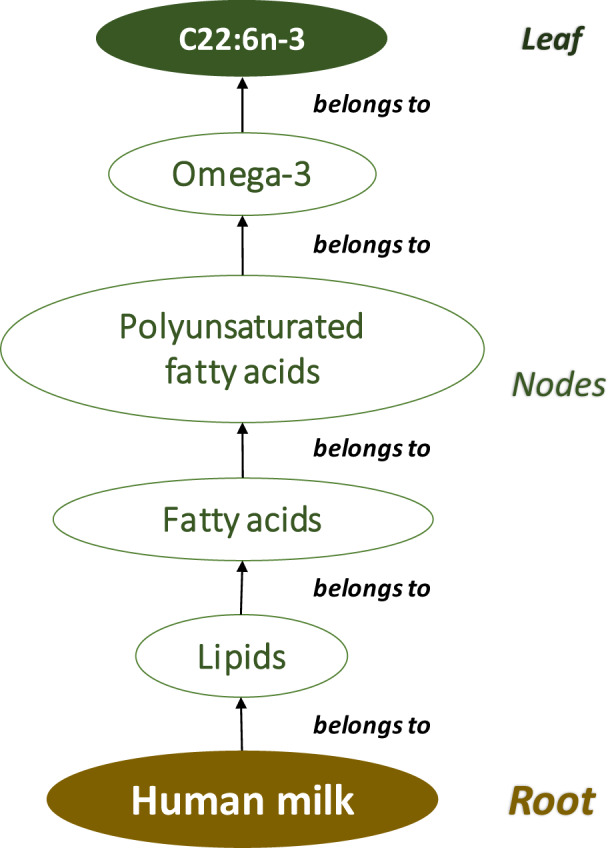
Tree-structure of components.

For compatibility, we fixed the “mass/volume milk” concentration of each biochemical component as the target response value. By “Component” we mean either a molecule or a group of molecules, such as say “linoleic acid”; or “fatty acid”. Both are “Components”, while the first is a special case of the second. Grouping like this follows a hierarchical tree structure as published data suggest (see Fig. 1). This way, not only the density of a particular molecule, but any components from the level next to the HM root, can be inputted.

Many authors only publish rescaled or derived values as components. Examples for this are the 2FL and 2FL/OS components (concentration of 2-fucosylated lactose and its proportion to that of the total oligosaccharides). To deal with such scenarios, we call a numerical value for 2FL as *direct*, while that for the 2FL/OS ratio as *indirect response*. We considered the explanatory and response variables as vectors, where each entry in the first one is a (mostly quantified) value on a specific condition that resulted in the response variables, in either direct or indirect form. Then a measurement for an indirect variable, such as 2FL/OS, is analogous to an implicit relationship between two mathematical variables. Similarly, a variable with the name “C18:1n-9 + C18:3n-3” indicates that the two fatty acids were measured together. So, the name of a variable may contain the “:” character to make it close to their biochemical notations as much as possible, as well as the “/” and “+” special characters, as mnemonic codes for derived variables.

The recorded values for these response variables are given in a so-called “extended numerical” format. By this, we mean that the inputted number can be supplied with its ± standard deviation or with an interval around it (like minimum-maximum, or quantile), both characterizing the uncertainty of the data. What is more, we differentiate between raw observations and estimations. Both can be inputted as response values, in the latter case with standard errors or confidence intervals. Finally, the response can be also dynamic, i.e. its temporal variation is stored in a table, and a pointer to the table is the inputted entry for the variable.

The condition fields do not necessarily hold only (extended) numerical values as above. They can be Boolean values or (a list of) categories, too. In the same way how a number belongs to an interval, a category value can belong to a group or to several groups. An example for this is the geographical region, indicating where an observation was made: the category group for China, for example can be either “Asia” or “FarEast”. Similar ambiguous definitions can occur say with Vitamin-D, by which typically we mean Vitamin-D3, but this is not necessarily stated in the publications explicitly. Therefore, an accurate analysis of the data may introduce a probabilistic weight when characterizing the HM components at molecular level.

The variety of the data is restricted by the significance of the conditions on which the publications report. For example, the HM composition is rarely studied as a function of the sex of the new-born, so there is no separate field for that explanatory variable in the database, but the sex is included in the cond_c variable that contains relevant infant characteristics.

The veracity is also affected by confusions on statistical/numerical concepts. For example, sometimes the standard deviation of the measured values is mistaken with the standard error of their mean. Several publications have drawn the attention on this^14,15^, but the mistake is still frequent. Similarly, either the publication or the person inputting the data may confuse quantiles (which is about the spread of the raw data), with confidence intervals (which is about the precision of the estimation). Whenever such errors are detected, we either correct them (if it is obvious) or mark them in the database (in less obvious situations).

## Workflow

**Fig. 2:**
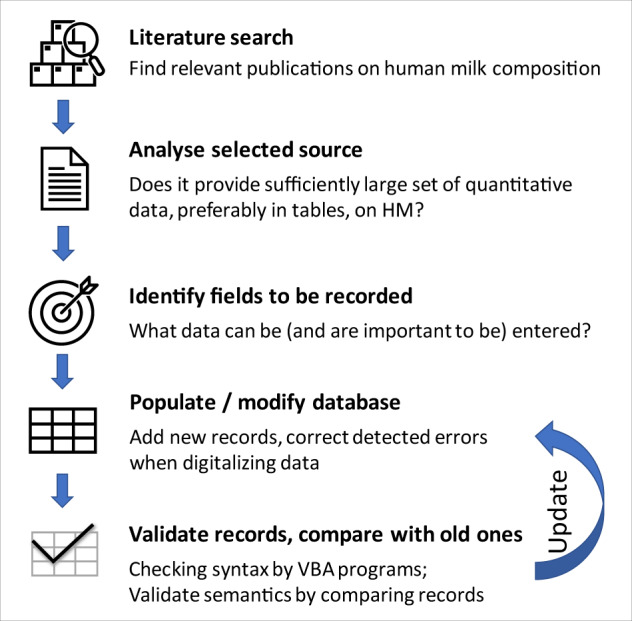
Workflow of building MilkyBase.

The workflow can be overviewed as shown in Fig. 2.

### Literature search

The publication search was partly manual, partly performed by FoodMine, a natural-language processing algorithm that finds papers on the chemical composition of a target food from PubMed^16^. The manual search used MeSH terms and Boolean operators in PubMed, with the following searching descriptor: (“human milk” OR “breast milk” OR “mothers’ milk”) AND (“nutrients” OR “components” OR “composition” OR “biochemical” OR “quantification” OR “bioactive”). The search was focused on, but not limited to, English language.

### Analyse source

The main selection criterium was quantitative data on the nutritional and/or non-nutritional components of HM. Priority was given to data (i) organized in a table format, in a systematic way; (ii) showing temporal variation (i.e., dynamic data); (iii) supplied with uncertainty quantification.

350 papers were selected by FoodMine and 201 were added from manual search. After elimination of irrelevant studies, a total of 365 potential papers were identified as suitable to enter the database. As of 1^st^ July 2022, MilkyBase contains data from 140 papers.

### Identify components

More than 600 (possibly derived) components have been identified so far, which can be either nodes or leafs of the tree-structured value set, or relationships between them. In this set, some individual molecules are represented both explicitly and implicitly (such as a specific fatty acid with unit g/litre of milk, also with a ratio to the total fatty acids, which is measured in gram. Taking out such duplicates, explicit measurements exist on ca 400 “genuine” components. Out of these, ca 50 are groups, i.e. they can be divided into either further groups or into molecules as the final leaves of the tree.

## Data Records

**Fig. 3:**
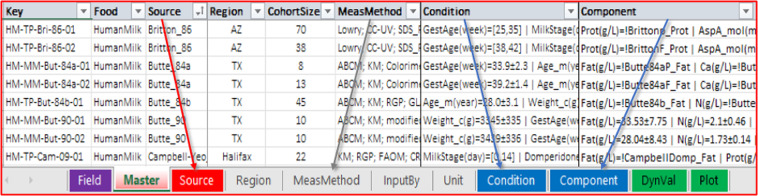
The MilkyBase database is a system of 10 linked tables. It The records of the core sheet are identified by a unique key and the possible values of a field are stored in the respective definition sheets with the same name.

The MilkyBase database is a system of connected tables represented by sheets in a single Microsoft Excel workbook (Fig. 3). Each record of its core (Master) sheet is identified by a unique key. Filling the source of the information, the geographic region of the measurement, the size of the cohort, the analytical method(s) measuring the component of interest in HM, as well as at least one condition and at least one response value are compulsory. The values in the Component and Condition fields can be “extended numerical” (e.g., numbers supplied with uncertainty quantification) as well as time-dependent series of numerical values, i.e., dynamic values. The syntax and the descriptions of the fields can be followed in sheets called “definition sheets”. These are also used by the “Syntax check” macro, which is part of the MBmacros.xlsm macro-enabled Excel workbook^17^, a collection of useful macros assigned to the database.

**Fig. 4:**
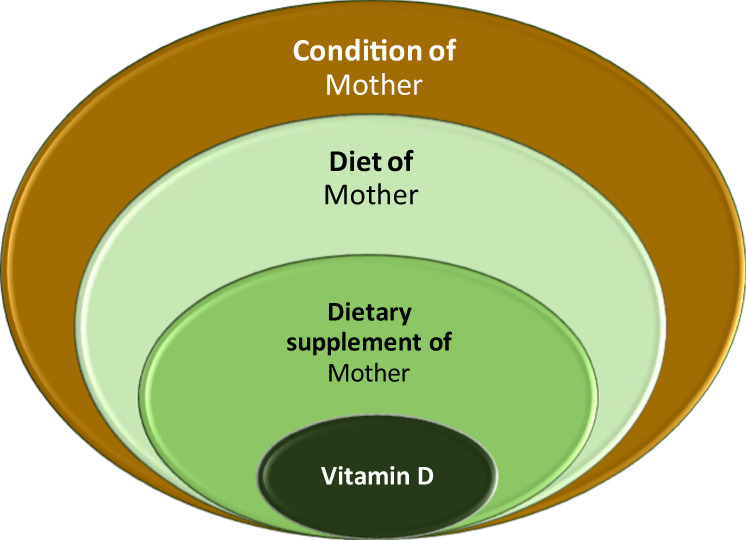
Example for the nested grouping of condition values.

The relationships between the entries follow a tree-structure as before (Fig. 4). For example, the entries in the Conditions field can be numerical, just as the Component field, but also categories, which are defined in a nested way. An example for this is “Vitamin D in the diet”, which belongs to the Diet group, which in turn belongs to the mother-related “condition_m” group.

A big part of the implicit responses are proportions, mostly the concentration of a specific fatty acid molecule compared to the total fatty acid. From these, the concentration of the fatty acid molecule in question can be estimated only if the total fatty acid is known. The same holds for the situation when a molecule is measured in molecular weight; this can be converted to concentration only if the mol-weight is known; these are given in a separate field of the Master sheet. Therefore, it is possible that a certain molecule is measured in 2-3 ways. Deducing all these duplicates, the final number of explicitly recorded concentrations of molecules is currently 326. The list is expected to constantly expand as new data are coming in.

The information belonging to the *CONDITION* field have been organised in a similar way. 60 variables are identified and put in 6 main groups. The details are provided in the description file MBdescription.pdf^17^.

The MilkyBase.xlsx and its technical description MBdescription.pdf as well as the mentioned macros provided in a file called MBmacros.xlsm, were deposited in Figshare^17^.

## Technical Validation

The database validation was helped by MS Excel VBA macros. The MBmacros.xlsm file containing them is available at the Figshare repository^17^.

It was straightforward to develop a “Syntax check” code but semantic check would require biochemical understanding. Various comparative plots were used to identify anomalies in the publications, such as wrong units, contradictions between figures and tables or misinterpreted data-scatter and uncertainty quantifications.

## Usage Notes

The presented MilkyBase database hosts records on milk composition in linked Excel tables. Its main novelty is the ontology that focusses on the effect of conditions under which the milk composition was measured, and the dynamics and uncertainity characteristics of these data, which will be entered in the explanatory and response fields. Its purpose is to provide a resource for researchers and a template for laboratories to put their own data into this format, thus initiating a knowledge-share following a kind of Wiki-philosophy.

Though the job of digitizing published data is rather laborious, as not everything can be automated, the main challenge in the development is its variety and veracity. “What to record” is a major decision and can be even biased.

It is impossible to totally automate the task of verification, either. Despite all the programming efforts, the task and responsibility must remain in the hands of the inputter and will remain dependent on human skill and expertise.

**Fig. 5:**
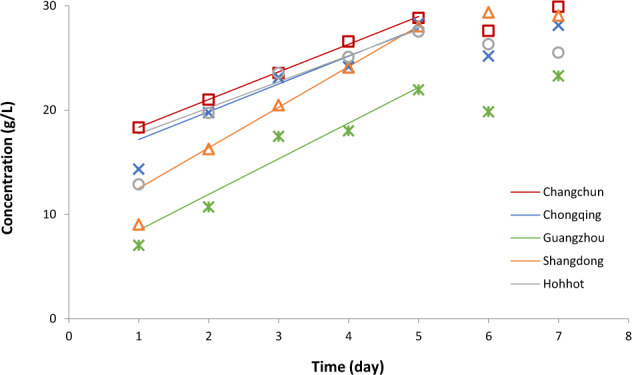
Temporal variation of fatty acid concentration in colostrum breast milk in five cities of China^19^. After the first day, the increase of the fatty acid is remarkably linear until day 5, with similar slopes, except in Shangdong (see the continuous lines, fitted to the data shown in respective colours). The fatty acid levels of breast milk (but not the rate of its increase) in Guangzhou are significantly different from those in the other four cities.

An example for the multivariate dynamic response inputted in a record is shown by Fig. 5. Such visualization is an aid to (i) recognize patterns and outliers in the data; (ii) identify data gaps; (iii) possibly identifying errors. For example this figure gives the idea, that the end of colostrum period can be defined as the time when the linear increase of fatty acid concentration is over.

**Fig. 6:**
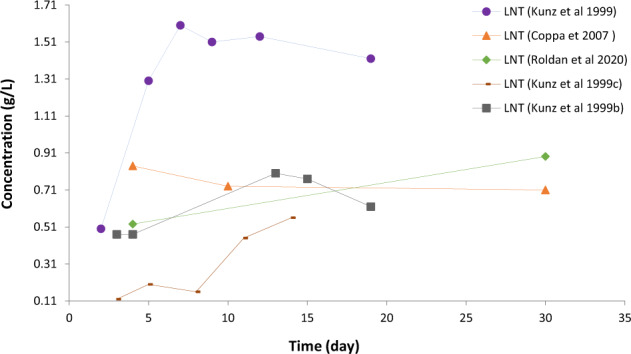
Visualisation gives ideas what relationships and patterns should be checked regarding the temporal variation of components.

Figure 6 compares the temporal variations of the concentration of Lacto-N-tetraose (LNT) in human milk as found by different authors. Here the observations of Kunz *et al*.^18^ show significant difference from other data, lending itself to an investigation what caused these differences.

MilkyBase demonstrates what benefits big data methods can bring for nutrition sciences. On a systematically organised database, users can run automated search and statistics that can help identifying data gaps (i.e., ideas for new research); finding mistakes in publications; and recognizing patterns, or possibly even model and optimize them for healthy infant and mother. A database like this needs to be of a relatively big volume (considering the complexity of the biochemical composition of milk), to get over a critical mass, from which we can consider the results as significant. Therefore, especially at the beginning of such database development, the amount of data that the authors make available in tables, plays a big role in the choice what papers should be digitized and recorded. Initially, the findings based on such database is inevitably more affected by what is *derivable* from the database, rather than what question is *desirable* to be solved by means of the database.

## Data Availability

MilkyBase.xlsx and its technical description MBdescription.pdf as well as the mentioned macros in an MBmacros.xlsm file, are available from the Figshare repository^17^.
